# Long-term outcomes of two-stage revision with positive cultures at reimplantation

**DOI:** 10.1007/s00402-026-06236-0

**Published:** 2026-02-26

**Authors:** Caterina Rocchi, Carmine Fabio Bruno, Rocco Cannata, Katia Chiappetta, Guido Grappiolo, Mattia Loppini

**Affiliations:** 1 Department of Biomedical Sciences, Humanitas University, Rozzano, Italy; 2 IRCCS Humanitas Research Hospital, Rozzano, Italy

**Keywords:** Periprosthetic joint infections, Two-stage revision, Total joint arthroplasty, Positive cultures

## Abstract

**Introduction:**

microbiological eradication after two-stage revision is not obtained in up to 18% of cases, yet the prognostic value of positive cultures at reimplantation remains controversial. The primary aim of the study was to evaluate outcomes of patients with positive cultures at second stage, identifying failure predictors. The secondary outcome was to compare reintervention-free survival.

**Materials and methods:**

this retrospective cohort study included patients treated using a two-stage protocol between 2016 and 2022. PJI was diagnosed using MSIS 2013 criteria, and treatment failure was defined according to a Delphi-based consensus. Cox regression analysis was employed to assess risk factors for failure, including Charlson Comorbidity Index (CCI); American Society of Anesthesiologists (ASA) score; age; time to reimplantation; joint; number of previous septic revisions; positive cultures number at reimplantation; a difficult to treat organism.

**Results:**

83 cases were reviewed (63 hips, 20 knees). The average interval between stages was 181 days. Over 6 years follow-up (FU), elevated BMI was the only significant predictor of failure (HR 1.19; 95% CI 1.02–1.39; *p* = 0.03). In contrast, positive cultures at reimplantation were not associated with an increased failure risk (*p* = 0.95), even in cases with multiple positive cultures (*p* = 0.72).

**Conclusions:**

elevated BMI at reimplantation was independently associated with subsequent failure. Clinical outcomes were not significantly associated with the presence or number of positive cultures, although smaller effects cannot be excluded given the limited sample size. These findings emphasize the importance of a patient-focused rather than culture-centered approach.

**Level of evidence:**

III.

## Introduction

### Background and current knowledge

Periprosthetic joint infections (PJI) remain one of the most devastating complications of total joint arthroplasty (TJA). Despite affecting only approximately 2% of patients, they represent a major cause of revision surgery, with a strong financial impact on healthcare systems worldwide [1–3]. As the number of joint replacements is projected to rise significantly, by up to 85% for primary procedures and 182% for revisions over the next decade, also the incidence of PJIs is expected to grow [4, 5]. Despite increasing clinical focus and a rapidly expanding body of literature, treatment outcomes have shown limited improvement over time [6]. 

Two-stage revision represents the standard of care to deal with critical PJI cases, particularly when facing soft tissue injury, unknown or multi-resistant pathogens, lack of access to appropriate antibiotics or lack of expertise of the surgical team [7]. This surgical technique involves removing the infected prosthesis, soft tissues debridement, and placement of an antibiotic-loaded temporary spacer with systemic antibiotic therapy. After presumed infection resolution, the surgeon re-evaluates the patient for the positioning of a permanent implant [7, 8]. 

### Controversies concerning positive cultures at reimplantation

A critical issue that could emerge at the time of reimplantation is the persistence of positive cultures. In fact, microbiological eradication at second stage is not obtained in 15 to 30% of cases [9, 10]. 

The prognosis for patients with positive cultures at reimplantation remains a controversial and underexplored issue, along with the minimum positive cultures number linked to a significant risk of infection recurrence and re-revision. Indeed, despite some studies considering single positive cultures as mere contaminants, others have evidenced that they might impact on future implant survival [11, 12]. The considerable incidence of positive cultures at reimplantation highlights the necessity to investigate further into their prognostic relevance.

Understanding the implications of positive cultures at reimplantation is essential, as certain pathogens, such as Gram-negative bacteria, streptococci, polymicrobial infections and resistant organisms are linked to higher failure and mortality rates [13, 14]. Additionally, these positive cultures must be interpreted with a patient-tailored approach, as some patient-related factors such as older age, American Society of Anesthesiology (ASA) score ≥ III, and McPherson host type C may contribute to poorer outcomes and correlate with persistent infection or inability to proceed to reimplantation [15, 16]. 

### Study rationale and aims

There is no consensus concerning the prognostic relevance of positive cultures at reimplantation. The primary aim of this study was to evaluate the outcome of patients undergoing two-stage revision for hip and knee PJIs with at least one positive culture at the time of reimplantation, identifying potential risk factors associated with failure. The secondary outcome was to determine the reintervention-free survival among patients with negative cultures at reimplantation and among those presenting with at least one positive culture.

## Materials and methods

### Study design

**Fig. 1:**
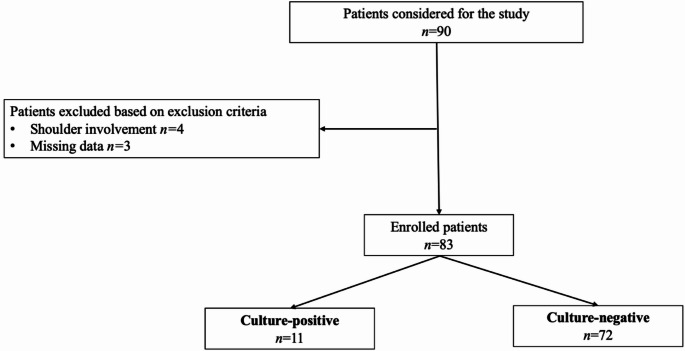
Patients’ inclusion flowchart

A retrospective observational study was conducted utilizing medical records from a registry of orthopaedic surgical procedures conducted at a high-volume referral center. The study protocol for the development of this registry was approved by the Institutional Ethical Committee (Approval number 618/17) and in strict accordance with the Helsinki Declaration. Patients who underwent hip or knee prostheses reimplantation between 2016 and 2022 were identified, employing the ICD-9-CM code 84.57 (removal of cement spacer) and excluding shoulder arthroplasty cases. Inclusion criteria comprised inpatients diagnosed with PJI who underwent two-stage total hip arthroplasty (THA) or total knee arthroplasty (TKA) revisions, with available histological records. Conversely, patients were excluded if they had missing anamnestic data or were under 18. PJI diagnosis has been formulated with the Musculoskeletal Infection Society (MSIS) 2013 criteria [17], and the definition of failure of treatment was performed with a Delphi-based consensus. Patients’ inclusion flowchart is shown in Fig. 1.

### Surgical technique and samples analysis

All the included procedures were conducted by experienced orthopaedic surgeons in a single referral center, using a posterolateral and median parapatellar approach for the hip and knee, respectively. The two-stage surgical management involved removing the infected prosthetic devices, debriding bone and soft tissues and positioning a temporary spacer. Static cemented gentamycin-loaded spacers were used for the hip, while both static spacers and the Hoffman technique were employed for the knee. Hip spacers were positioned with proximal neck cementation, guaranteeing mechanical stability and ease of removal. The Hoffman technique involved placement of an articulated construct featuring an isolated femoral cruciate-retaining component and a cemented polyethylene tibial unit. This approach was preferred for patients with preserved bone stock and stable collateral ligaments, when an articulated spacer allowing temporary joint motion could be safely maintained during the interval stage.

Empirical antibiotic therapy (piperacillin/tazobactam and vancomycin, vancomycin and levofloxacin, or cefazolin) was given after explant surgery until microbiological results were available; the therapeutic regimen and duration was then adapted to detected pathogens. After infection resolution, confirmed through normalized serum C-reactive protein levels in three consecutive measurements (< 0.5 mg/dL), the surgeon re-evaluated each patient for the positioning of a permanent implant.

No preoperative antibiotic prophylaxis was administered, and a 12-days washout period was observed prior to samples collection. Synovial fluid and periprosthetic tissue were collected for each patient for microbiological analysis, both at time of explant surgery and reimplantation. Multiple biopsies (5–7 samples for each patient) were performed intraoperatively to increase the sensitivity, and to distinguish potential contamination from actual pathogens. Each sample was stored in a dedicated container to avoid cross-contamination. Three sets of cultures were obtained intraoperatively for each patient: periprosthetic tissue samples, synovial fluid, and prosthesis sonicate. In addition to the classical culture, each sample underwent a culture for anaerobic bacteria for 14 days and a second for filamentous fungi. Prosthetic devices were subjected to sonication in Ringer’s solution (50 Hz for 5 min) to detach the biofilm; then, the resulting fluid was sent to microbiological analysis. Leukocyte count was performed on synovial fluid using manual microscopy. Before analysis, samples were gently mixed to reverse sedimentation. A sample with fewer than 500 cells/µL was considered negative.

### Postoperative rehabilitation

A structured rehabilitation protocol was adopted after each of the two stages of THA and TKA revision. The recovery regimen began in the immediate postoperative period and was tailored to the physical status of each patient, taking spacer-related precautions after the first stage. Early mobilization exercises, including passive and active mobilization for range of motion (ROM) recovery and ambulation with assistive devices, were initiated after both stages, to prevent joint stiffness and muscle atrophy development. As the healing progressed, the physiotherapeutic program evolved to include balance training and strengthening exercises. The latter were focused on the quadriceps, hamstrings, gluteal muscles, and calf muscles for the knee, and on abductor muscles, iliopsoas, and quadriceps for the hip. Partial weightbearing was recommended until reimplantation after the first stage, and for the first 3–4 weeks postoperatively after the second.

### Outcomes measurement

Both patient-related and procedure-related factors were considered, to evaluate their potential as predictors of failure of two-stage revisions. Patients’ comorbidities were assessed with the Charlson Comorbidity Index (CCI) [18]. This scoring system assesses patient survival by considering several concurrent factors, including age, myocardial infarction, heart failure, cerebrovascular disease, dementia, chronic pulmonary disease, connective tissue disease, liver disease, diabetes, hemiplegia, renal disease, active cancer, HIV/AIDS.

A quantitative assessment of functional joint recovery was made by comparing the pre and postoperative values of Harris Hip Score (HHS) [19] and Knee Society score (KSS) [20], two specific questionnaires employed to measure clinical outcomes. The two scores combine both questions directed to patients (pain, walking support, presence of limp, walking distance, ability to walk stairs, put on shoes and sit) and a clinical evaluation of the ROM of the involved joint.

### Data analysis

Cox regression analysis was performed to assess failure predictors. Variables included in the analysis were CCI, ASA score, smoker status, diabetes, rheumatoid arthritis, involved joint, sex, age, body mass index (BMI), time to reimplantation (TTR), previous septic revisions, positive cultures number at reimplantation, synovial fluid positivity, a difficult to treat organism. Cultures positivity was examined both as a binomial variable (“positive cultures”) and as specific subcategories (“synovial fluid positivity,” “single positive culture,” and “multiple positive cultures”), to investigate the impact of the number and type of positive cultures on clinical outcomes. Results were reported as Hazard Ratio (HR) with 95% confidence interval (95% CI); *p* < 0.05 was considered as statistically significant for both the univariate and multivariate models.

Discrete variables were described as numbers and percentages, whereas for continuous variables the mean and standard deviation were obtained, with range if necessary. The analysis of categorical variables was made using Pearson’s *Chi*-square or Fisher’s exact tests, whereas continuous variables were evaluated using the 2-sample t-test or the Wilcoxon rank-sum tests. The Wilcoxon signed-rank test was employed to detect changes of continuous variables from preoperative values to the last follow-up, because of the presence of non-Gaussian data distribution (Shapiro-Wilk test, *p* < 0.05).

Re-intervention free survival was calculated from surgery to re-intervention date using the Kaplan-Meier method, and reported as percentage and 95% CI at 1 to 6 years. A post-hoc assessment of detectable effect size was conducted employing the Schoenfeld/Hsieh-Lavori method for Cox regression [21, 22]. 

All analyses were performed with STATA17. Graphs were generated using GraphPad PRISM10.

## Results

A total of 83 patients were considered eligible for the study, including 63 (75.9%) two-stage hip revision arthroplasties and 20 (24.1%) two-stage knee revision arthroplasties. 36 patients (43.37%) were men, and average age at operation was 67.9 ± 11.62 years old. The average time to reimplantation (TTR) was 181 days (range, 21-1051). One patient (1.2%) underwent spacer exchange in between the two stages, resulting from infection persistence. In 11 patients (13.25%), at least one positive culture was identified at reimplantation, and 1 (9.09%) of them subsequently failed, compared with 6 patients (8.33%) who were culture-negative (HR: 0.94, *p* = 0.95). The same initially infecting organism was isolated at reimplantation in 2 out of 11 culture-positive patients (18.18%). Additionally, within the culture-positive patients’ group, 2 (18.18%) presented negative cultures at the time of prosthesis explant and spacer placement. No demographic characteristic could be significantly associated with positive cultures occurrence (*p* > 0.05). Pathogens identified at second stage were methicillin-resistant Staphylococcus Epidermidis (36.36%, *n* = 4), methicillin-sensitive Staphylococcus Aureus (9.09%, *n* = 1), methicillin-sensitive Staphylococcus Epidermidis (9.09%, *n* = 1), methicillin-resistant Staphylococcus Capitis (9.09%, *n* = 1), Enterobacter Cloacae (9.09%, *n* = 1), Staphylococcus Pasteuri (9.09%, *n* = 1), Candida Albicans (9.09%, *n* = 1), and a polymicrobial infection with Enterococcus Faecalis, Proteus Mirabilis and Staphylococcus Epidermidis (9.09%, *n* = 1). Demographic analysis is shown in Table 1.

**Table 1:** Patients’ demographic data

Demographic characteristics	Culture-negative (*n* = 72)	Culture-positive (*n* = 11)	Total (*n* = 83)	*p*-value
Joint (hip)	53 (73.71%)	10 (90.9%)	63 (75.90%)	0.21
BMI (mean ± SD)	29.1 ± 4.8	28.6 ± 4.7	29.02 ± 4.78	0.75
Sex (M)	30 (41.67%)	6 (54.54%)	36 (43.37%)	0.42
Active Smoker	16 (22.22%)	5 (45.45%)	21 (25.3%)	0.09
Rheumatoid Arthritis	2 (2.78%)	0	2 (2.41%)	0.58
Diabetes Mellitus type II	12 (16.67%)	2 (18.18%)	14 (16.87%)	0.9
Age (mean ± SD)	67.74 ± 11.61	69 ± 12.3	67.9 ± 11.6	0.74
Time to Reimplantation (TTR)				0.32
< 100 days	12 (16.67%)	4 (36.36%)	16 (19.28%)	
100–200 days	40 (55.56%)	4 (36.36%)	44 (53.01%)	
200–300 days	15 (20.83%)	3 (27.27%)	18 (21.68%)	
> 300 days	5 (6.94%)	0	5 (6.02%)	
Previous septic revision	9 (12.5%)	2 (18.18%)	11 (13.25%)	0.61
Charlson Comorbidity Index				0.76
High Comorbidity Profile (CCI > 2)	57 (79.17%)	9 (81.81%)		
Low Comorbidity Profile (CCI ≤ 2)	15 (20.83%)	2 (18.18%)		
ASA score				0.07
ASA score I	8 (11.11%)	1 (9.09%)	9 (10.84%)	
ASA score II	42 (58.33%)	5 (45.45%)	47 (56.63%)	
ASA score III	22 (30.56%)	4 (36.36%)	26 (31.32%)	
ASA score IV	0	1 (9.09%)	1 (1.23%)	
Cultures at Explant				0.16
Culture-negative	21 (29.17%)	2 (18.18%)	23 (27.71%)	
MSSE	4 (5.56%)	1 (9.09%)	5 (6.02%)	
MRSE	10 (13.89%)	1 (9.09%)	11 (13.25%)	
MSSA	8 (11.11%)	0	8 (9.64%)	
MRSA	4 (5.56%)	1 (9.09%)	5 (6.02%)	
Enterococcus Faecalis	6 (8.33%)	2 (18.18%)	8 (9.64%)	
Staphylococcus Lugdunensis	5 (6.94%)	0	5 (6.02%)	
Polymicrobial Infection	4 (5.56%)	1 (9.09%)	5 (6.02%)	
CONS	2 (2.78%)	0	2 (2.41%)	
Staphylococcus Caprae	2 (2.78%)	0	2 (2.41%)	
Staphylococcus Capitis	1 (1.39%)	1 (9.09%)	2 (2.41%)	
Pasteurella Multocida	1 (1.39%)	0	1 (1.2%)	
Pseudomonas Aeruginosa	1 (1.39%)	1 (9.09%)	2 (2.41%)	
Bacillus Cereus	1 (1.39%)	0	1 (1.2%)	
Streptococcus Bovis	1 (1.39%)	0	1 (1.2%)	
Streptococcus Sanguinis	1 (1.39%)	0	1 (1.2%)	
Salmonella Spp.	0	1 (9.09%)	1 (1.2%)	
Technique at first stage				0.58
Gentamycin-loaded spacer	70 (97.22%)	11 (100%)	81 (97.59%)	
Hoffman technique	2 (2.78%)	0	2 (2.41%)	

*M* male sex,* BMI* body mass index,* ASA* American society of anesthesiology score,* CCI* Charlson comorbidity index,* MSSE* Methicillin-sensitive staphylococcus Epidermidis,* MRSE* Methicillin-resistant staphylococcus epidermidis,* MSSA*Methicillin-sensitive staphylococcus aureus,* MRSA* Methicillin-resistant staphylococcus aureus,* CONS* Coagulase-negative staphylococci

The only variable significantly associated with the treatment failure was higher BMI (HR 1.19; 95% CI 1.02–1.39; *p* = 0.03). Cultures positivity did not significantly increase failure risk (*p* = 0.95), and neither did the presence of multiple positive cultures (*p* = 0.23). Multivariate analysis is shown in Table 2.

**Table 2:** Cox regression analysis between culture negative and positive group

Joint (hip/knee)	Univariate HR	*p*-value	Multivariate HR	*p*-value
	0.51	0.50		
Positive Cultures	0.94	0.95		
Antibiotic resistance	1.75	0.63		
Multiple positive cultures	1.5	0.72		
One positive culture	NA	0.51		
Synovial positivity	NA	0.48		
Sex	1.08	0.92		
Age	0.97	0.58		
BMI	**1.18**	**0.03**	**1.19**	**0.03**
ASA score	1.07	0.91		
CCI	1.2	0.83		
Smoker status	1.07	0.94		
Diabetes Mellitus type II	0.88	0.9		
Rheumatoid Arthritis	NA	0.675		
Previous septic revision	1.19	0.88		
Time to Reimplantation	1	0.11		

*M* male sex,* BMI* body mass index,* ASA* American society of anesthesiology score,* CCI* Charlson comorbidity index,* HR* hazard ratio,* NA* not applicable due to limited statistical power

**Fig. 2:**
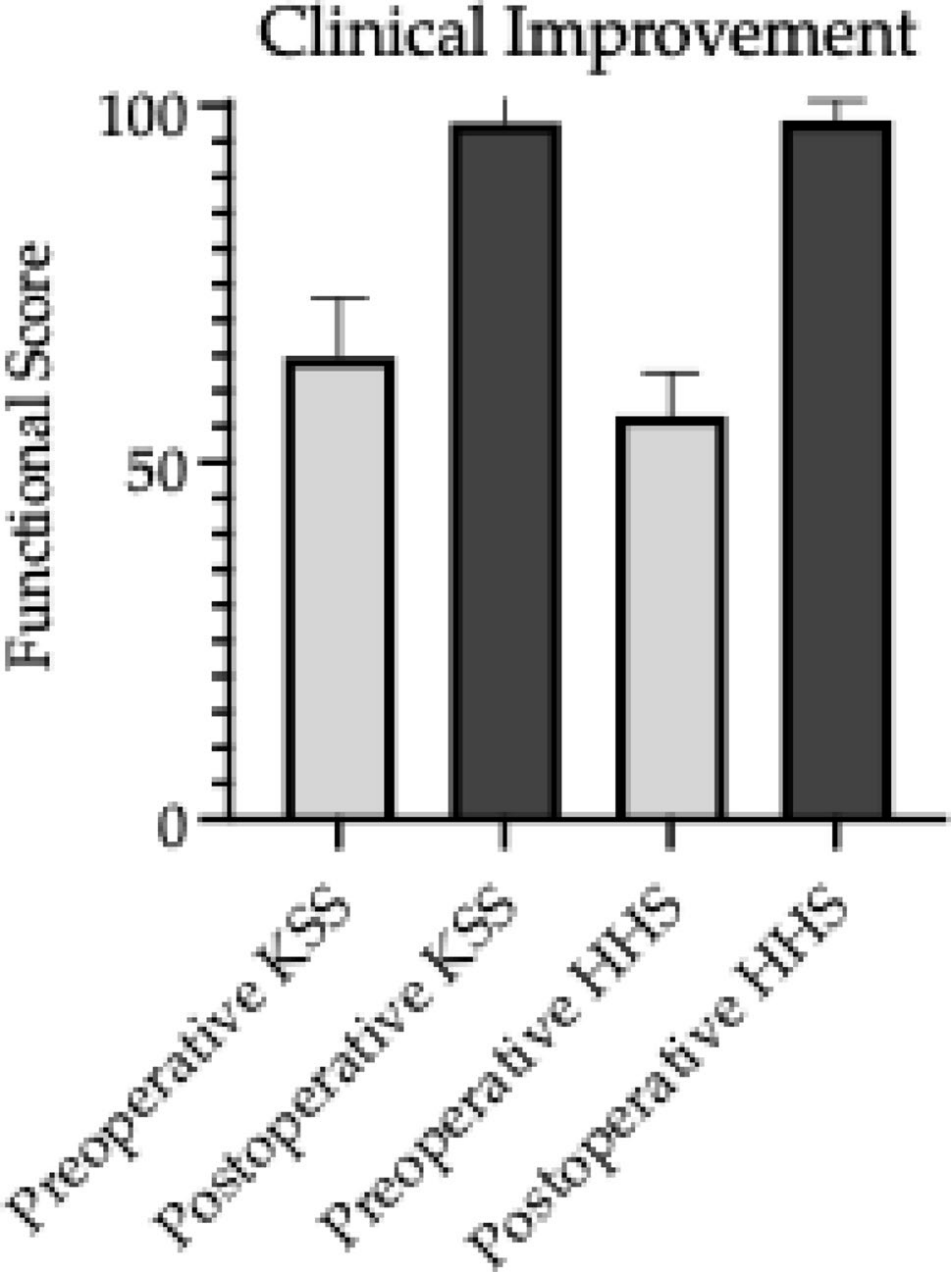
Improvement in functional status before and after surgery, as measured by the Harris Hip Score (HSS) and Knee Society Score (KSS)

The mean HHS improved from 56.62 ± 6.01 (range 40–72, *p* < 0.01) to 97.98 ± 2.79 (range 81–100, *p* < 0.01). Similarly, a significant increase was seen in KSS values, raising from average preoperative values of 65.52 ± 7.9 (range 50–80, *p* < 0.01) to postoperative 98.21 ± 3.31 (range 86–100, *p* < 0.01). Enhancement of patients’ functional status before and after surgery is shown in Fig. 2.

With a mean follow-up of 77.61 (2-115) months, 6 (8.33%) out of 72 hips in the culture-negative group underwent re-revision surgery: one because of mechanical dislocation of the acetabular component (1.39%), another one because of recurrent dislocation (1.39%), a third one because of aseptic dislocation of the femoral stem (1.39%), and three because of infection (4.17%). Conversely, one patient (9.09%) among those presenting positive cultures at reimplantation underwent re-revision during the follow-up period because of aseptic mechanical dislocation. Early and late postoperative complications are listed in Table 3.

**Table 3:** Early (≤ 30 days) and late (> 30 days) postoperative complications. Early wound-related complications were managed with surgical site revision

Early Complications (≤ 30 days)	Culture-Negative (*n* = 72)	Culture-Positive (*n* = 11)	Total (*n* = 83)	*p*-value
	4 (5.56%)	0	4 (4.8%)	0.42
Implant Dislocation	1 (1.39%)	0	1 (1.2%)	
Postoperative fracture	1 (1.39%)	0	1 (1.2%)	
Surgical Site Revision	2 (2.78%)	0	2 (2.41%)	
Late Complications (> 30 days)	7 (9.72%)	1 (9.09%)	8 (9.64%)	0.95
Diaphyseal Fracture	1 (1.39%)	0	1 (1.2%)	
Aseptic Mechanical Dislocation	2 (2.78%)	1 (9.09%)	3 (3.6%)	
Reinfection	3 (4.17%)	0	3 (3.6%)	
MRSE	1 (1.39%)	0	1 (1.2%)	
MSSA	1 (1.39%)	0	1 (1.2%)	
Culture-negative	1 (1.39%)	0	1 (1.2%)	
Recurrent Dislocation	1 (1.39%)	0	1 (1.2%)	

Prosthetic dislocation was treated with closed reduction. Lastly, one patient sustained a Vancouver type B2 periprosthetic fracture following a fall on postoperative day 13, which was also treated with open reduction and internal fixation (ORIF) using a plate and screws. Concerning late complications, a femoral diaphyseal fracture was treated with ORIF with plate and screws. Aseptic dislocations of the stem or acetabular component and reinfection cases were treated with re-revision surgery, and defined as failure cases

*MRSE* Methicillin-resistant staphylococcus epidermidis,* MSSA* Methicillin-sensitive staphylococcus aureus

**Fig. 3:**
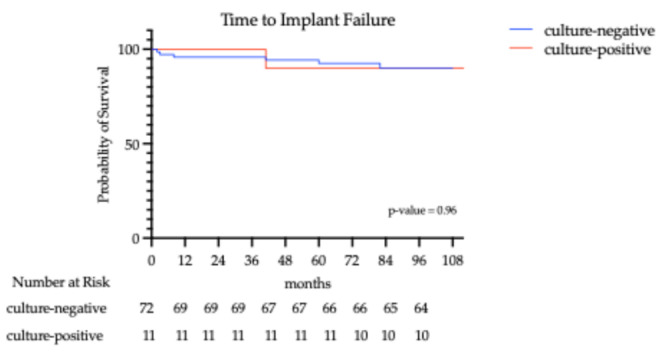
Kaplan-Meier curve of reintervention-free survival (culture-positive vs. culture-negative cases)

According to the Kaplan-Meier curve, survival remained high in both groups throughout the follow-up period. At 12 and up to 36 months, survival remained near 100% in both groups. However, beyond 36 months, both groups exhibited a slight decline in survival. By the final follow-up (up to approximately 115 months), survival had dropped to 91.60% (95% CI:78–96%) in the culture-negative and 90.91% (95% CI: 57–99%) in the culture-positive group. Although patients with negative cultures appeared to have slightly higher survival over time, the difference was not statistically significant (*p* = 0.96). Two patients, both members of the culture-negative group, died during the follow-up period due to unrelated clinical conditions, with their implants unrevised. The first one deceased because of a stroke at 47 months follow-up, and the second of unknown unrelated causes after 94 months. The Kaplan-Meier curve of reintervention-free survival is presented in Fig. 3.

## Discussion

### Prognostic significance of positive cultures at reimplantation

The main finding of the present study was that the presence of positive cultures at reimplantation in two-stage THA and TKA revisions did not correlate with poor subsequent outcomes. In fact, contrary to previous assumptions, culture positivity at reimplantation was not predictive of implant failure (HR 0.94, *p* = 0.95). In the past, the presence of positive cultures at reimplantation has been associated with increased risk of revision compared to their absence (41.1 vs. 14.8% respectively, OR 4.58). However, the odds of reinfection could be significantly decreased with proper antibiotic treatment [9]. While persistent culture positivity has traditionally been considered a major contraindication to reimplantation, our findings support an expanding body of research suggesting that their prognostic value may be limited [23]. Indeed, culture positivity has been associated with comparable failure risk in the setting of two-stage revisions for chronic PJIs (*p* > 0.05) [24]. Furthermore, in our work, the presence of multiple (≥ 2) positive cultures did not reflect an increased failure risk (HR = 2.95, *p* = 0.23). This is in contrast with previous findings, which reported poorer clinical outcomes in patients with two or more positive cultures, compared to those with negative or isolated positive cultures [25]. Discrepancies in pathogen profiles and management protocols, such as antibiotic stewardship, timing of reimplantation, or surgical technique, may partially account for the differing results. Additionally, although in our study the presence of two or more positive cultures did not reach statistical significance in predicting reinfection, the increasing HR may suggest a subtle emerging tendency towards increased risk that may not have been detected due to limited sample size.

Unfortunately, the effects of isolated synovial fluid positivity and a single positive culture could not be properly investigated within our cohort due to their limited occurrence. The prognostic significance of positive cultures quantity should be further examined to define the minimum threshold associated with increased failure risk.

### Patient- and treatment-related predictors of failure

The only significant independent predictor of failure identified in this study was BMI. Indeed, both in univariate and multivariate Cox regression models, higher BMI was associated with an increased failure risk, with a HR of 1.19 (95% CI: 1.02–1.39, *p* = 0.03). This finding is consistent with previous literature, underscoring that obesity is a key modifiable risk factor in TJA outcomes. A meta-analysis including 15 studies indicated a threefold increase in the risk of septic revision failure among obese and morbidly obese patients (BMI ≥ 30 kg/m² and ≥ 40 kg/m² respectively) [26]. Similarly, elevated BMI has been associated with decreased five-year survival after two-stage revision arthroplasty [27]. Lastly, the increased adipose tissue in obese patients can impair wound healing and reduce soft tissue integrity, contributing to longer postoperative recovery and increased risk of complications. An enhancement of perioperative protocols, including proper glycemic control, skin decontamination and ultraclean operative environment, is essential to decrease the risk of complications in this category of patients [28]. Furthermore, the fact that BMI is a relevant but modifiable risk factor for revision emphasizes the importance of weight control in arthroplasty patients.

Taken together, our findings underscore that positive cultures at reimplantation should not be examined as an isolated factor but rather interpreted within a broader clinical context. Factors such as BMI, overall health status, and soft tissue quality may exert a stronger influence on long-term outcomes than culture positivity alone.

Antibiotic resistance was not found to be a significant failure predictor within our cohort (HR: 1.75, CI: 0.21–14.29, *p* = 0.63). However, the wide CI observed suggest that this variable might be clinically relevant in a larger sample.

Only 16 patients (19.28%) were reimplanted within 100 days, and longer TTR was not associated with failure risk (*p* = 0.32). Although TTRs reported in literature range from a few to several hundred days, their prognostic role remains controversial. Indeed, evidence suggesting an advantage for shorter TTRs is often biased by confounding by indication, as patients selected for early reimplantation often present with lower virulence and favorable clinical profiles [29]. Conversely, procedures included in our study were performed in a tertiary-level referral center, and several cases involved persistent or multisite infections. Our findings emphasize that favorable outcomes may depend more on case-by-case clinical evaluation than on arbitrary temporal thresholds.

### Strengths and limitations

The strengths of this study lie in the long-term follow-up period, detailed assessment of microbiological data, and clinically applicable findings. Conversely, its main limitations arise from its retrospective design and consequently the potential for recall bias. Moreover, there was limited incidence of positive cultures within our cohort, and a larger sample size would have increased the generalizability of the results. Given the low number of failure events despite the long-term follow-up period, the post-hoc power estimation indicated < 10% power to detect anything but very large differences between groups (HR > 6). This means that smaller clinically relevant effects may not have been detected. Our findings should therefore be interpreted as informative rather than definitive, opening the way to wider, multicentric analyses to understand the implications of reimplanting culture-positive patients.

## Conclusion

Body Mass Index at reimplantation was independently associated with subsequent failure. Patients with one or more positive cultures at reimplantation did not appear to have worse outcomes than culture-negative counterparts, although the low number of failures limited the ability to detect smaller but clinically meaningful differences. These findings emphasize the importance of a patient-centered rather than culture-centered approach, that goes beyond microbiological results to include individual risk factors.

## Data Availability

Data supporting reported results can be found in a repository (Zenodo).
